# The association of serum magnesium and mortality outcomes in heart failure patients: A systematic review and meta-analysis

**DOI:** 10.1097/MD.0000000000005406

**Published:** 2016-12-16

**Authors:** Teeranan Angkananard, Thunyarat Anothaisintawee, Sudarat Eursiriwan, Oleg Gorelik, Mark McEvoy, John Attia, Ammarin Thakkinstian

**Affiliations:** a Section for Clinical Epidemiology and Biostatistics, Faculty of Medicine, Ramathibodi Hospital, Mahidol University, Bangkok; b Division of Cardiovascular Medicine, Department of Medicine, Faculty of Medicine, HRH Princess Maha Chakri Sirindhorn Medical Center, Srinakharinwirot University, Ongkharak, Nakhon Nayok; c Department of Family Medicine, Faculty of Medicine, Ramathibodi Hospital, Mahidol University; d Cardiology Unit, Department of Pediatrics, Faculty of Medicine Vajira Hospital, Navamindradhiraj University, Bangkok, Thailand; e Department of Internal Medicine “F”, Assaf Harofeh Medical Center (Affiliated to the Sackler Faculty of Medicine, Tel Aviv University, Tel Aviv, Israel), Zerifin, Israel; f Center for Clinical Epidemiology and Biostatistics, The School of Medicine and Public Health, the University of Newcastle, Newcastle, NSW, Australia.

**Keywords:** heart failure, magnesium, meta-analysis, mortality, systematic review

## Abstract

**Background:**

Low serum magnesium (Mg) has been independently shown to increase risk of heart failure (HF), but data on the association between serum Mg concentration and the outcome of patients with HF are conflicting. The purpose of this systematic review and meta-analysis was to estimate the prognostic effects of hypermagnesemia and hypomagnesemia on cardiovascular (CV) mortality and all-cause mortality (ACM) of patients with HF.

**Methods:**

Relevant studies were identified from Medline and Scopus databases. Included and excluded criteria were defined. Effects (i.e., log [risk ratio [RR]]) of hypomagnesemia and hypermagnesemia versus normomagnesemia were estimated using Poisson regression, and then a multivariate meta-analysis was applied for pooling RRs across studies. Heterogeneity was explored using a meta-regression and subgroup analysis.

**Results:**

On analysis, 7 eligible prospective studies yielded a total of 5172 chronic HF patients with 913 and 1438 deaths from CV and ACM, respectively. Most participants were elderly men with left ventricular (LV) ejection fraction ≤40%. Those patients with baseline hypermagnesemia had a significantly higher risk of CV mortality (RR, 1.38; 95% confidence interval [CI], 1.07–1.78) or ACM (RR, 1.35; 95% CI, 1.18–1.54) than those with baseline normomagnesemia. However, baseline hypomagnesemia was not associated with the risk of CV mortality (RR, 1.11; 95% CI, 0.79–1.57) and ACM (RR, 1.11; 95% CI, 0.87–1.41). A subgroup analysis by Mg cutoff suggested a dose–response trend for hypermagnesemia effects, that is, the pooled RRs for CV mortality were 1.28 (95% CI, 1.05–1.55) and 1.92 (95% CI, 1.00–3.68) for the cutoff of 0.89 to 1.00 and 1.05 to 1.70 mmol/L, respectively.

**Conclusion:**

The present systematic review and meta-analysis suggested that, in HF patients, hypermagnesemia with serum Mg ≥ 1.05 mmol/L was associated with an increased risk of CV mortality and ACM but this was not observed for hypomagnesemia. This finding was limited to the elderly patients with chronic HF who had reduced LV systolic function.

## Abstract 2

Supplemental Digital Content is available in the text

## 1 Introduction

Heart failure (HF) is a major public health burden. It was estimated that 5.7 million US adults aged ≥20 years were diagnosed with HF between 2009 and 2012,^[1]^ and the new HF cases will increase to about 870,000 annually.^[1,2]^ One-month readmission after hospitalization is close to 25%, and 1-year mortality rate is as high as 22%.^[3]^ HF also carries a significant economic burden worldwide, with an overall predicted cost in the United States of $69.7 billion in 2030. Several biomarkers are potentially useful for prediction of clinical outcomes in management of acute and chronic HF, for example, serum sodium and natriuretic peptides (B-type natriuretic peptide [BNP] and N-terminal pro-BNPs [NT-proBNPs]). Measurement of natriuretic peptides is currently recommended for establishing prognosis or disease severity in chronic HF.^[4]^ However, the cost-effectiveness of NT-proBNP–guided therapy is still questionable in some patients, particularly in the elderly with several comorbidities.^[5]^ Furthermore, measurement of NT-proBNP is not available as a routine test in developing countries. Therefore, new prognostic biomarkers which are easily measured and inexpensive should be explored.

Magnesium (Mg) is the second most abundant intracellular cation and the fourth most abundant cation in the body.^[6]^ It is an important dynamic ion for transcellular transport which involves several efflux and influx systems. With its vasodilatory, anti-inflammatory, anti-ischemic, and antiarrhythmic properties, it is considered an important nutrient which might be a potentially useful therapeutic agent for cardiovascular (CV) diseases including hypertension, atherosclerosis, acute myocardial infarction (MI), arrhythmia, and left ventricular (LV) hypertrophy.^[7,8]^

Hypomagnesemia is a frequent electrolyte disorder in HF patients occurring either as an isolated disturbance or with other acid–base and electrolyte abnormalities. This condition may be associated with potentially life-threatening arrhythmia, in which effective correction is beneficial in patients with HF.^[9]^ In addition, low serum Mg has been independently shown to increase risk of HF,^[10]^ but this effect has not been replicated by recent studies.^[11,12]^

Therefore, we performed a systematic review and meta-analysis of prospective studies aiming at estimating the prognostic effects of hypomagnesemia and hypermagnesemia on CV death and all-cause mortality (ACM) of patients with HF.

## 2 Methods

### 2.1 Search strategy

Relevant studies were identified from Medline and Scopus databases from inception until June 2015. Searching was performed using the following search terms: “heart failure, diastolic”, diastolic failure, “heart failure, systolic”, systolic failure, congestive heart failure, congestive cardiomyopathy, myocardial failure, low ejection fraction, magnesium, serum magnesium, blood magnesium, total magnesium, magnesium blood level, Magnesium/∗blood, normomagnesemia∗, hypermagnesemia∗, hypomagnesemia∗, death, mortality, fatality, survive∗, sudden death, cardiovascular mortality, rehospitalization∗, readmission, admission, and hospitalization∗.

Search strategies for each database are described in Appendix (refer to Supplemental Digital Content 1, which demonstrates search terms and search strategy used for Medline and Scopus database). Reference lists of included studies were explored to identify additional studies.

### 2.2 Selection of studies

Identified studies were selected based on their titles and abstracts, and on the full papers if a decision could not be made. Inclusion criteria were as follows: any prospective human studies published in English; studied in adults; had serum Mg concentration available; Mg concentration was measured as intracellular free Mg or extracellular ionized Mg; reported ACM or CV mortality; and provided sufficient data for pooling, that is, number of subjects between Mg groups and HF outcomes. Studies with insufficient data were excluded after 3 failed attempts to contact authors.

### 2.3 Data extraction

All data were independently extracted by 2 reviewers (the lead and second authors). Discrepancies were resolved through discussion with the senior author. The following characteristics of studies and patients were extracted: setting of HF, follow-up time, type of HF, sex, mean age, body mass index (BMI), underlying diseases (i.e., diabetes, hypertension, chronic kidney disease [CKD], history of MI, LV ejection fraction [LVEF]), the New York Heart Association (NYHA) functional classification, baseline diuretic used, measurement of serum Mg concentration, its cutoff, and outcomes. Cross-tabulated data at a certain time between serum Mg groups and CV mortality/ACM were extracted for pooling. When these data were not available, risk ratio (RR)/hazard ratio along with 95% confidence interval (CI) was extracted instead, and if that was not available, data were extracted from Kaplan–Meier curves.

### 2.4 Risk of bias assessment

Study quality was assessed by the same 2 independent authors (the lead and second authors) using validated quality criteria in prognostic studies (QUIPS tool).^[13]^ Discrepancies were made by consensus with the senior author. Six domains were considered: study participation (5 items), study attrition (4 items), prognostic factor measurement (5 items), outcome measurement (3 items), study confounding (6 items), and statistical analysis and reporting (3 items). Each item was rated as yes, no, or unsure if there was low, high risk, or there was insufficient information (refer to Table, Supplemental Digital Content 2, which illustrates quality bias assessment, stratified by each domain). The overall validity of each domain was graded as low, moderate, and high risk of bias if all, 2/3, and less than or equal to 1/3 of items were rated as yes, respectively.

### 2.5 Studied factor

The factor of interest was serum Mg concentration measured in mmol/L. The mg/dL and mEq/L units were converted to mmol/L by multiplying by 0.41 and 0.50, respectively. Serum Mg concentration was categorized into 3 groups (i.e., hypomagnesemia, hypermagnesemia, and normomagnesemia) according to original cutoffs of individual studies.

### 2.6 Outcomes of interest

The outcomes of interest were CV mortality and ACM. CV mortality was defined according to the original studies including sudden death, pump failure, MI, and stroke. ACM was defined as composite CV mortality and other deaths. CV mortality was used instead of ACM if studies did not report ACM.

### 2.7 Statistical analysis

A summary of cross-tabulated data for hypomagnesemia, hypermagnesemia, and normomagnesemia versus CV mortality and ACM were expanded to individual-patient data. Effects (i.e., log [RR]) of hypomagnesemia and hypermagnesemia versus normomagnesemia were then estimated using Poisson regression by fitting Mg groups on outcomes.^[14]^ A multivariate meta-analysis was next applied for pooling RRs across studies, and subject-study correlation was accounted for using Riley method.^[15]^

The Cochrane *Q* statistics and *I*^2^ statistic were applied to assess heterogeneity. Possible sources of heterogeneity including Mg cutoff, age, gender, BMI, prior MI, diabetes, hypertension, CKD, type of HF, NYHA class, and LVEF were explored by a meta-regression and subgroup analysis.

Publication bias was explored using a funnel plot and Egger test. All analyses were performed using STATA software (version 14.0, Stata Corp, College Station, TX). A 2-sided test with *P* value <0.05 was considered statistically significant except for the test of heterogeneity, in which a *P* value <0.10 was applied.

This study used the secondary data for analysis. Therefore, this study was not submitted to the institutional review board for ethical approval.

## 3 Results

**Figure 1:**
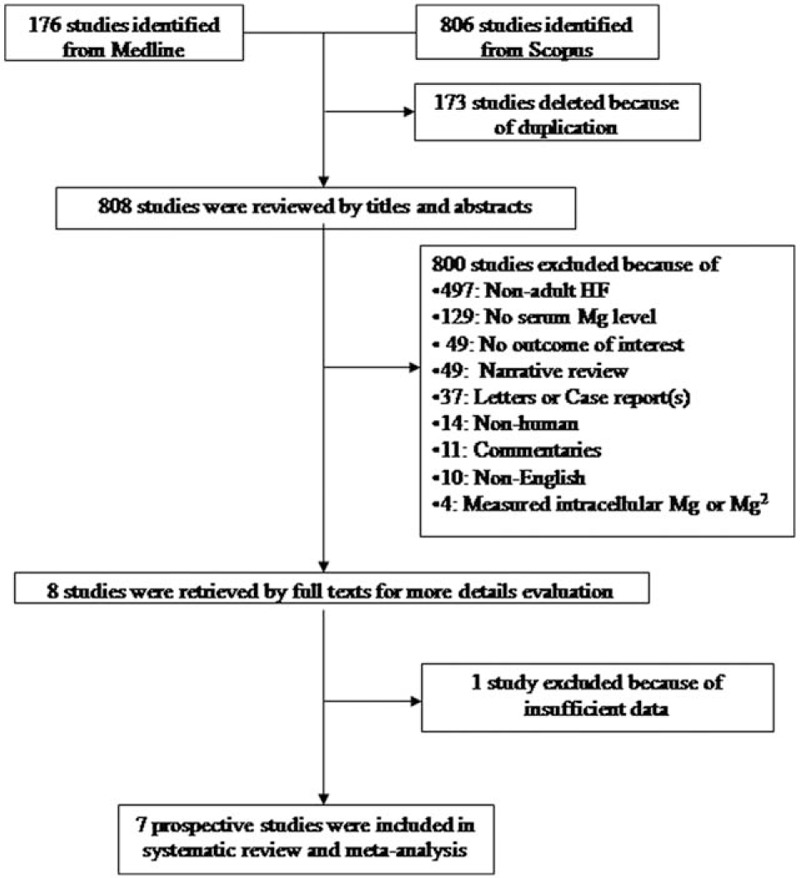
Flowchart of study selection.

A total of 808 studies were identified from Medline and Scopus databases, of which only 8 studies^[11,12,16–21]^ were eligible for pooling (Fig. 1). One study^[21]^ was excluded because of insufficient data; 7 studies^[11,12,16–20]^ comprising 5172 chronic HF with reduced ejection fraction patients were finally included. Characteristics of the 7 included studies are described in Table 1. Among them, 4 studies were prospective cohorts and 3 studies were randomized controlled trials using Mg data at baseline. The follow-up times ranged from 9.9 to 43 months. ACM and CV mortality were reported in 7 and 6 studies, respectively. Four studies were conducted in the United States and Canada, 2 in Europe, and the last in Israel. Participants were predominately male and elderly (mean age 55.4–77.2 years). Percents of patients with ischemic heart disease, LVEF ≤ 40%, and CKD ranged from 22.1% to 70.4%, 68.3% to 100%, and 23.2% to 46.4%, respectively. Most studies^[11,12,16–19]^ recruited HF patients with NYHA classes III and IV. Studies used standard analytic approaches for serum Mg determination, that is, colorimetric assay, atomic absorption spectroscopy, or autoanalyzers and bioassays on Architect platform (Abbott, Wiesbaden, Germany).

**Table 1:**
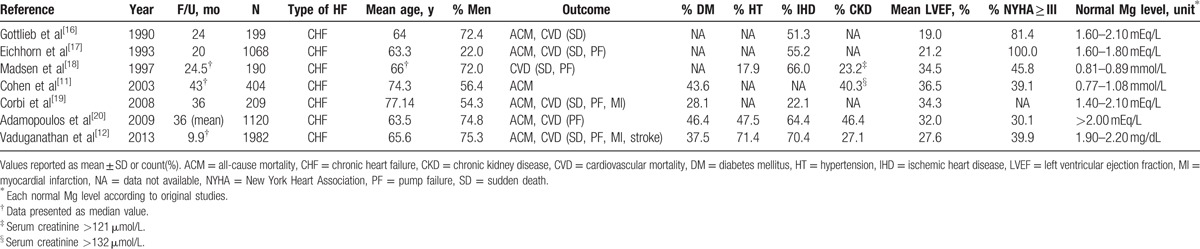
Characteristics of included studies.

For assessing the effect of serum Mg concentration on mortality, 5 studies^[11,12,16–18]^ compared hypomagnesemia and hypermagnesemia with normomagnesemia, one study^[19]^ compared only hypermagnesemia with normomagnesemia, and one study compared only hypomagnesemia with normomagnesemia.^[20]^ Old age and CKD were common features in HF patients with hypermagnesemia (refer to Table, Supplemental Digital Content 3, which illustrates patients’ characteristics grouped according to serum Mg concentration). Only 3 studies^[12,19,20]^ reported diuretic use ranging from 50% to 97.8% of patients.

### 3.1 Risk of bias assessment

**Figure 2:**
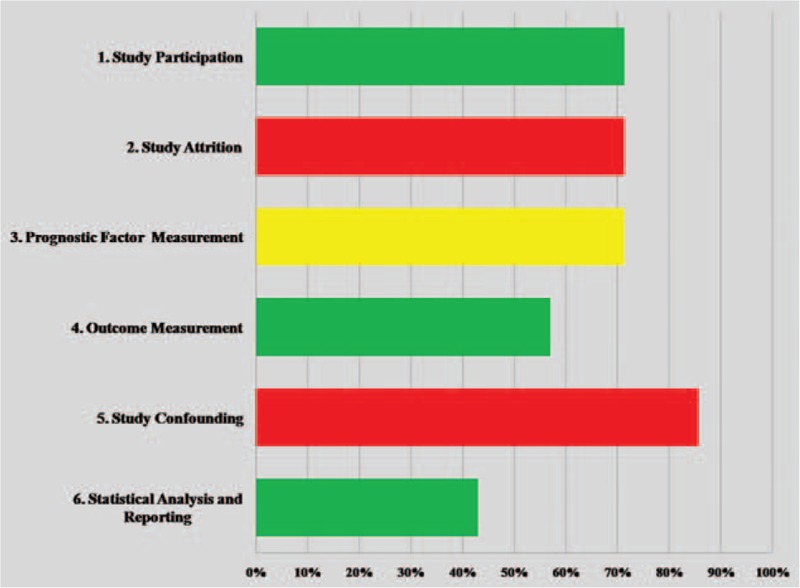
Risk of bias assessment (green = low risk of bias, yellow = moderate risk of bias, red = high risk of bias).

Risk of bias assessment is presented in Fig. 2. All 7 studies were graded as low risk of bias for the domains of study participation and outcome measurement. However, all studies were rated high risk of bias for study confounding and attrition, and intermediate risk for prognostic factor measurements.

### 3.2 CV mortality

**Figure 3:**
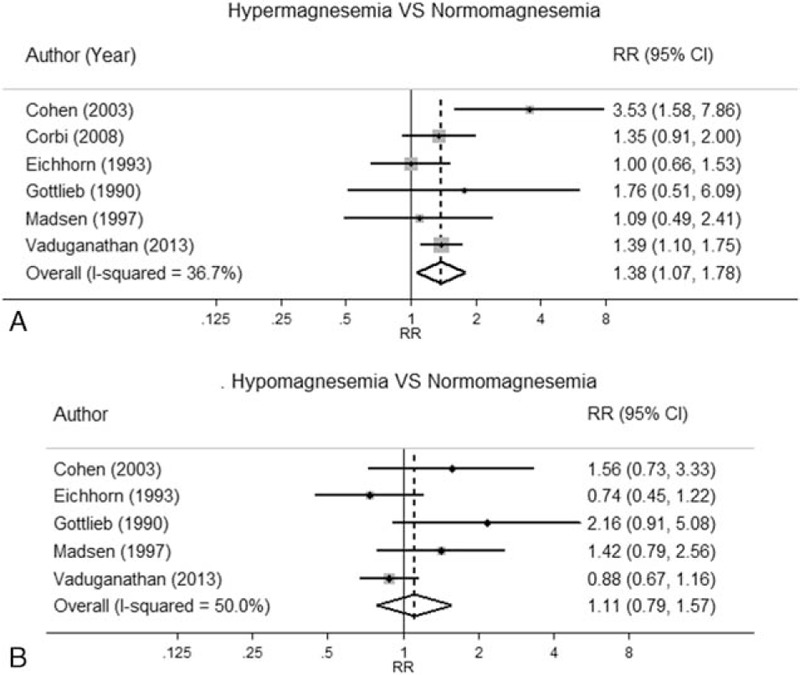
Association between abnormal serum magnesium concentration and cardiovascular mortality.

Risks of CV mortality among hypomagnesemia, hypermagnesemia, and normomagnesemia groups for each study are described in Table 2. Use of cutoff thresholds for hypomagnesemia and hypermagnesemia varied from 0.74 to 1.00 and 0.89 to 1.09 mmol/L, respectively. One study^[20]^ assessed Mg effect by comparing hypomagnesemia with normomagnesemia (≤1 vs >1 mmol/L), in which the comparator group (i.e., normomagnesemia) was very differently defined from other studies. This study was therefore excluded from the main analysis. Effects of hypermagnesemia (n = 6) and hypomagnesemia (n = 5) versus normomagnesemia were estimated and described (Fig. 3). The effects were moderately heterogeneous for both hypomagnesemia and hypermagnesemia with *I*^2^ of 50% (*Q* test = 7.99, *P* = 0.09) and 36.7% (*Q* test = 7.90, *P* = 0.16), respectively.

**Table 2:**
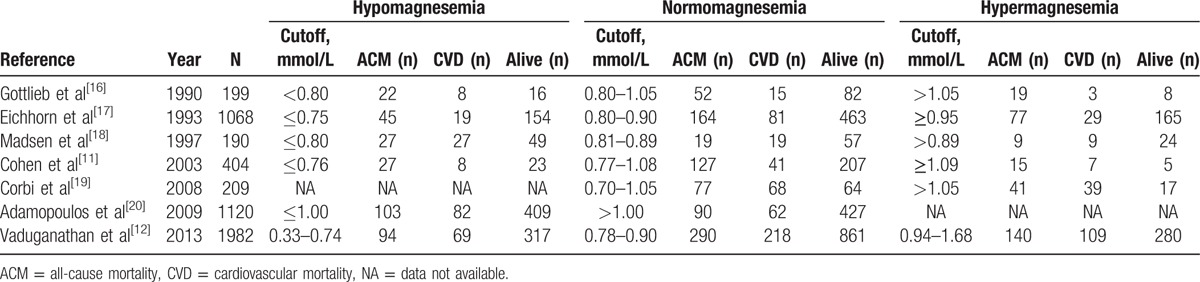
Cross-tabulation data between magnesium groups versus all-cause and cardiovascular mortality.

The pooled RRs were 1.11 (95% CI, 0.79–1.57) and 1.38 (95% CI, 1.07–1.78) for hypomagnesemia and hypermagnesemia compared with normomagnesemia, respectively.

This suggested that HF patients with hypermagnesemia were about 38% more likely to die from a CV event than those with normomagnesemia.

**Figure 4:**
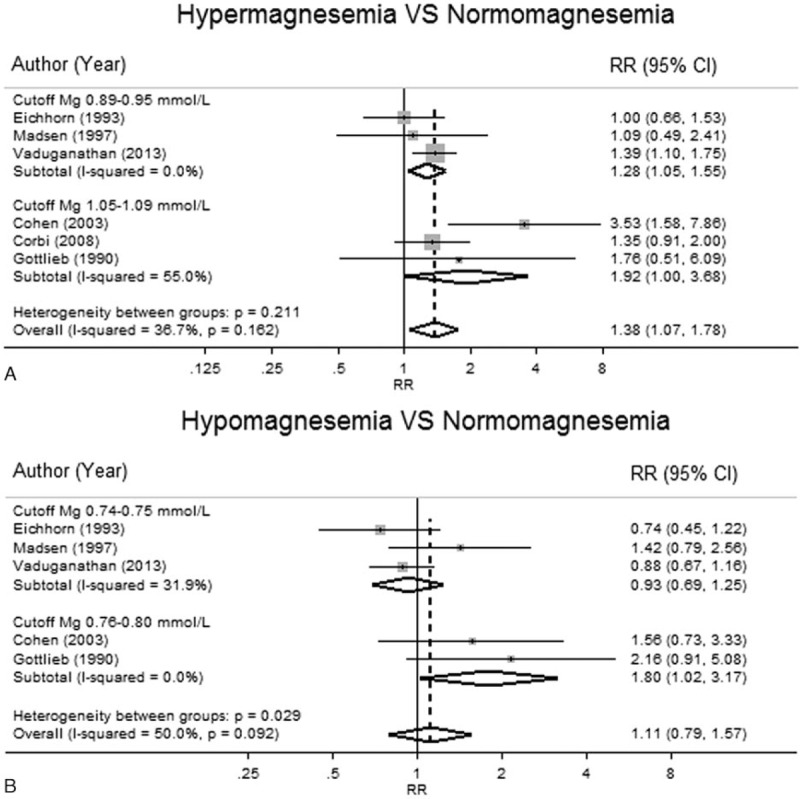
Subgroup analysis by magnesium cutoff level on cardiovascular mortality.

A subgroup analysis by Mg cutoff suggested a dose–response trend for hypermagnesemia effects (Fig. 4), that is, the pooled RRs for CV mortality were 1.28 (95% CI, 1.05–1.55) and 1.92 (95% CI, 1.00–3.68) for the cutoff of 0.89 to 1.00 and 1.05 to 1.70 mmol/L, respectively. For hypomagnesemia, the pooled RRs for CV mortality were 0.85 (95% CI, 0.67–1.08) and 1.60 (95% CI, 1.07–2.41) for the cutoff of 0.70 to 0.75 and 0.76 to 0.80 mmol/L, respectively.

Pooling of 2 studies,^[12,19]^ with high use of diuretics (ranging from 50% to 97.6%) yielded a pooled RR of 1.75 (95% CI 1.03–2.98) (refer to Figure, Supplemental Digital Content 4, which illustrates subgroup analysis by percentages of diuretic use). In addition, subgroup analysis by mean age (≤70 vs >70 years), follow-up time (≤2 vs >2 years), NYHA class of HF (II–IV vs only III–IV), and use of diuretic were examined, but none of them was identified as a source of heterogeneity (refer to Table, Supplemental Digital Content 5, which demonstrates effects of hypermagnesemia vs normomagnesemia on CV mortality: subgroup analyses by factors).

Publication bias was further assessed for both hypomagnesemia and hypermagnesemia using funnel plots and Egger test, indicating no evidence of publication bias (refer to Figure, Supplemental Digital Content 6, which illustrates funnel plots for CV mortality).

### 3.3 ACM

**Figure 5:**
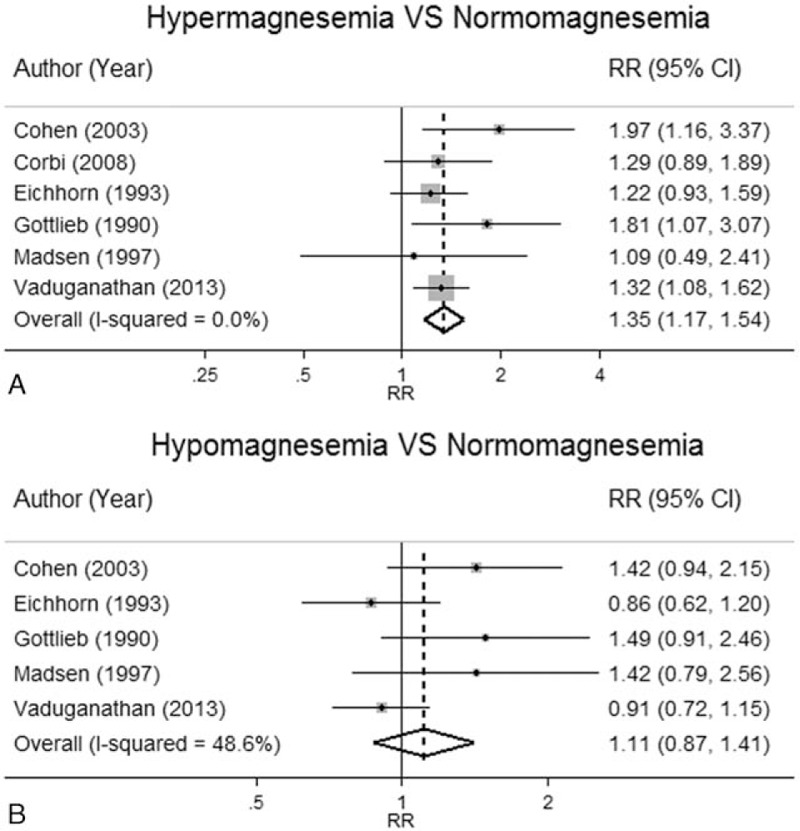
Association between abnormal serum magnesium concentration and all-cause mortality.

Cross-tabulated data for Mg groups and ACM are shown in Table 2. Cutoff thresholds for hypomagnesemia and hypermagnesemia varied from 0.74 to 1.00 and 0.89 to 1.09 mmol/L, respectively. One study^[20]^ was again excluded as mentioned previously. Effects of hypermagnesemia (n = 6) and hypomagnesemia (n = 5) versus normomagnesemia were homogeneous (*I*^2^ = 0%, *Q* test = 4.07, *P* = 0.539) and moderately heterogeneous (*I*^2^ = 48.6%, *Q* test = 7.78, *P* = 0.100), respectively. The corresponding pooled RRs were 1.35 (95% CI, 1.18–1.54) and 1.11 (95% CI, 0.87–1.41) (Fig. 5). This suggested that HF patients with hypermagnesemia are about 35% more likely to die from any causes than those with normomagnesemia. Funnel plot and Egger test suggested no evidence of publication bias (refer to Figure, Supplemental Digital Content 7, which illustrates funnel plot for ACM). A subgroup analysis of hypomagnesemic effect on CV mortality and ACM could not be performed because there were not enough studies for pooling (n = 5).

## 4 Discussion

Our systematic review and meta-analysis suggested that HF patients with hypermagnesemia (serum Mg level ≥0.89 mmol/L) were approximately 38% and 35% more likely to die from CV diseases and all causes than those with normomagnesemia, whereas no significant effects of hypomagnesemia (serum Mg level ≤0.74 mmol/L) were observed. The risk of CV mortality almost doubled when hypermagnesemia ranged over 1.05 to 1.09 mmol/L.

Our results were similar to that observed in a recent study of dysmagnesemia in hospitalized patients,^[22]^ in whom serum Mg ≥ 0.96 mmol/L was associated with worse hospital mortality. In this study, hypermagnesemia was notably high (31.5% of total 65,974 patients) and was found commonly in those with CV disease, whereas hypomagnesemia was common in patients with hematologic and oncological disorders. However, our study could not observe an effect of hypomagnesemia on CV mortality or ACM despite previous studies observing an inverse association between both serum and dietary Mg and total CV events risk.^[23,24]^ These studies included individuals without prevalent CV disease at baseline and examined ischemic heart disease as the major outcome. This implied that high serum Mg concentration was a protective factor for CV disease. Paradoxically, data from our study suggested that it was associated with worse clinical outcomes in individuals with HF. Mg has also been recognized as having an antiatherosclerotic effect; a recent meta-analysis^[23,24]^ found a significant inverse association between serum Mg concentrations and the risk of total CV events in general patients. This might be explained by Mg retarding arterial calcification.^[25]^ Mg can theoretically interfere with arterial calcification by indirect actions via phosphate binding in the intestinal lumen, systemic effects on CKD-mineral and bone disorders associated factors, and direct actions at the level of vascular tissues. In addition, mild hypermagnesemia may have a protective vascular effect, whereas moderate hypermagnesemia reduces osteoid formation and causes parathyroid hormone suppression, leading to vascular calcifications.^[25]^

Adverse effects of hypermagnesemia in HF patients might be explained as follows: first, altered ion channel properties, sometimes induced by an altered oxido-redox state, may lead to proarrhythmic conditions and worse prognosis in subjects with normal hearts.^[26,27]^ As in earlier rat model studies, Mg ions compete with calcium ion-activating and ion-inactivating sites on the type II isoform ryanodine receptor channels in cardiac myocytes; thus, hypermagnesemia may cause impairment of both cardiac systolic contraction and diastolic relaxation.^[28]^ In the case of failing heart subjects, these abnormalities may be augmented by structural cardiac alterations. Second, hypermagnesemia appears to impair the release of acetylcholine and decreases motor end-plate sensitivity to acetylcholine in muscles. It might induce serious arrhythmia (bradycardia, prolonged PR, QRS, QT interval, and complete heart block), vasodilation, and myocardial depression which result in hypotension. Also, the prolonged corrected QT interval (QTc > 440 ms) is important in HF patients as a strong predictor of both pump failure and sudden death.^[29]^ Third, old age and renal impairment are associated with hypermagnesemia,^[30]^ and both are already known to be associated with mortality in HF patients. Thus, hypermagnesemia might directly influence mortality or it might be a mediator of those factors. Lastly, the high prevalence of hypomagnesemia in congestive HF subjects, which ranges from 7% to 52%,^[31]^ may cause physicians to have more awareness and lead to aggressive correction of this condition for prevention of its adverse effects, that is, cardiac arrhythmia, congestive HF, and other CV events than hypermagnesemic individuals. This intensive approach could explain the better prognosis in hypomagnesemic HF patients compared with their hypermagnesemic counterparts.

The findings from this review might be useful in routine clinical practice, because serum Mg concentration is a routine laboratory test recommended as an initial test for patients presenting with HF.^[4]^ Our data suggest that a serum Mg concentration higher than 0.89 mmol/L in elderly patients with low LVEF might be used for prognosis and for guided therapy in the chronic HF setting. In addition, these data might be useful for monitoring those patients who receive Mg supplementation.

Our study has some strengths and limitations. To the best of our knowledge, this is the first systematic review and meta-analysis to address the association between serum Mg concentration and clinical outcomes in patients with HF. The analysis was based on prospective studies with relatively large sample sizes, and these studies minimized the chance of selection or recall bias affecting the results. A multivariate meta-analysis was applied to simultaneously pool effects of hypomagnesemia and hypermagnesemia. Finally, our meta-analysis did not demonstrate publication bias, although the number of studies is low. Nevertheless, there were some limitations of our meta-analysis. Most studies recruited patients with poor prognosis, that is, included elderly patients with reduced LV systolic function (LVEF ≤ 35%). Therefore, the applicability of our results might be limited to older patients with impaired LV systolic function. In addition, the effect of hypermagnesemia might be confounded by other variables. Low Mg levels may reflect aggressive use of diuretics, and hence the better prognosis. Working on aggregated data did not allow us to adjust for all known confounders. Although the pooled data of diuretic use was done from only 2 studies as mentioned above, the effect of hypermagnesemia was still similar to the overall pooling. Therefore, use of diuretic might have less influence on effect of hypermagnesemia. However, hypermagnesemia itself might directly increase mortality or it might be due to the effect of underlying conditions (e.g., CKD, advanced age, etc.).

Currently, the use of implantable cardioverter-defibrillator therapy is recommended for primary prevention in patients 3 to 9 months from the initial diagnosis of nonischemic cardiomyopathy who still have significant LV dysfunction and HF symptoms.^[32]^ Cardiac resynchronization therapy can also improve cardiac function and structure in symptomatic chronic HF patients with optimal medical treatment, severely depressed LVEF (i.e., ≤35%) and complete left bundle branch block, reduce mortality and hospitalization.^[33]^ A recent study demonstrated that telemonitoring for HF patients with a cardiac resynchronization therapy implantable cardioverter defibrillator may reduce HF hospitalizations.^[34]^ Data for both therapies are crucial and may affect prognosis, but these data were not available and could not be extracted.

For establishing prognosis in chronic HF, the ratio of insulin-like growth factor-I serum concentrations and growth hormone and natriuretic peptides have been widely studied.^[35]^ The data for natriuretic peptides were reported in only one of the included studies^[12]^; this demonstrated an association with hypomagnesemia (serum Mg 0.8–1.8 mEq/L) and hypermagnesemia (serum Mg 2.3–4.1 mEq/L). However, its correlation with mortality was not reported. Recently, microRNAs have emerged as a biomarker of HF patient, which could predict responsiveness of cardiac resynchronization therapy in advanced HF patients with dyssynchrony.^[36–38]^ Future studies using individual patient data should be conducted to assess the causal pathway effect of hypermagnesemia on HF progression.

## 5 Conclusion

The present systematic review and meta-analysis suggests that in HF patients hypermagnesemia with serum Mg ≥ 1.05 mmol/L was associated with an increased risk of CV mortality and ACM but this was not observed for hypomagnesemia. This finding was limited to the elderly patients with chronic HF who had reduced LV systolic function.

## Acknowledgments

The authors are grateful to Dr Sasivimol Rattanasiri, Dr Sakda Arj-Ong Vallipakorn, Pawin Numthavaj, MD, and staff of the Section for Clinical Epidemiology and Biostatistics, Faculty of Medicine Ramathibodi Hospital, Mahidol University, for their cooperation in the initial step of searching for the publications for this study.

## Supplementary Material

Supplemental Digital Content
