# Supplementary material for: The association of serum magnesium and mortality outcomes in heart failure patients: A systematic review and meta-analysis
Source: Medicine (Baltimore). 2016 Dec 16;95(50):e5406. doi: 10.1097/MD.0000000000005406 (PMC5268025; doi:10.1097/MD.0000000000005406)

SUPPLEMENTAL CONTENT

Supplemental Digital Content 1. Appendix

A: Search terms and search strategy used for Medline database

((((((((((((magnesium) OR "serum magnesium") OR "blood magnesium") OR "total magnesium") OR "magnesium blood level") OR "Magnesium/*blood")) OR normomagnesemia*) OR hypermagnesemia*) OR hypomagnesemia*)) AND ((((((((((readmission) OR admission) OR rehospitalization*) OR hospitalization*) OR "cardiovascular mortality") OR "sudden death") OR survive*) OR fatality) OR death) OR mortality)) AND ((((((((("heart failure") OR "heart failure"[MeSH Terms]) OR "heart failure, systolic") OR "congestive cardiomyopathy") OR "cardiac failure") OR "low ejection fraction") OR "heart failure, diastolic") OR "congestive heart failure") OR "myocardial failure")

B: Search terms and search strategy used for Scopus database

((TITLE-ABS-KEY (**"heart failure"**) OR TITLE-ABS-KEY (**"systolicfailure"**) OR TITLE-ABS-KEY (**"diastolic failure"**) OR TITLE-ABS KEY (**"myocardial failure"**) OR TITLE-ABS-KEY (**"low ejectionfraction"**))) AND ((TITLE-ABS-KEY (**magnesium**) OR TITLE-ABS-KEY (**hypomagnesemia**) OR TITLE-ABS-KEY (**hypermagnesemia**) OR TITLE-ABS-KEY (**normomagnesemia**) OR TITLE-ABS-KEY (**[mg^{2+}]**) OR TITLE-ABS-KEY (**[mg^{2+}]**) OR TITLE-ABS-KEY (**"Mg ion"**))) AND ((TITLE-ABS-KEY (**mortality**) OR TITLE-ABS-KEY (**fatality**) OR TITLE-ABS-KEY (**hospitalization**) OR TITLE-ABS-KEY (**admission**) OR TITLE-ABS-KEY (**death**) OR TITLE-ABS-KEY (**survival**) OR TITLE-ABS-KEY (**rehospitalization**) OR TITLE-ABS-KEY (**readmission**)))

Supplemental Digital Content 2. Quality bias assessment, stratified by each domain

Domain	Issues for Consideration	Rating of Reporting						
		Gottlieb, et al. 1990	Eichhorn, et al. 1993	Madsen, et al. 1997	Cohen, et al. 2003	Corbi, et al. 2008	Adamopoulos, et al. 2009	Vaduganathan, et al. 2013
1. Study Participation								
1.1 Source of target population	The source population is adequately described, including who the target population is, when (time period of study), where (location), how (description of recruitment strategy) and period of recruitment.	Unsure	Yes	Yes	No	Yes	Yes	Yes
1.2 Method used to identify population	The sampling frame and recruitment are adequately described, including methods to identify the sample sufficient to limit potential bias.	No	Yes	Yes	No	Yes	Yes	Yes
1.3 Adequacy of population	Eligible population is adequate for the study.	Yes	Yes	Yes	Yes	Yes	Yes	Yes
1.4 Inclusion and Exclusion criteria	Inclusion and exclusion criteria are adequately described.	No	Yes	Yes	Unsure	Yes	Yes	Yes

1.5 Baseline Characteristics	The baseline study population is adequately described. Description of population would include clinical characteristics of individual (e.g. age, sex, CAD risk factors, NYHA functional class, LV ejection fraction, etiology of HF and baseline medication)	Yes	Yes	Yes	No	Yes	Yes	Yes
Summary study participation	The study sample represents the population of interest on key characteristics, sufficient to limit potential bias of the observed relationship between PF and outcome.	High	Low	Low	High	Low	Low	Low

Domain	Issues for Consideration	Rating of Reporting						
		Gottlieb, et al. 1990	Eichhorn, et al. 1993	Madsen, et al. 1997	Cohen, et al. 2003	Corbi, et al. 2008	Adamopoulos, et al. 2009	Vaduganathan, et al. 2013
2. Study Attrition								
2.1 Proportion of baseline sample available for analysis	Proportion of study sample completing the study and providing outcome data is adequate	Yes	Yes	Yes	Unsure	Yes	Yes	Yes
2.2 Attempts to collect data on participants who dropped out	Describe about the attempts to collect data on participants who dropped out of the study	No	No	Unsure	No	No	No	No
2.3 Reasons and potential influence of participants lost to follow-up	Reasons for loss to follow-up are provided	Yes	No	Yes	No	No	No	No
	Adequate description of participants lost to follow-up	Yes	No	Yes	No	No	No	No

	Small number of lost to follow-up, <10%	Yes	Yes	Yes	Unsure	Yes	Yes	Yes
2.4 Outcome and prognostic factor information on those lost to follow-up	Participants lost to follow-up are adequately described for characteristics of individual (e.g. age, sex, CAD risk factors, NYHA functional class, LV ejection fraction, etiology of HF and baseline medication) which there are no important differences between participants who completed the study and those who lost to follow-up	Unsure	Unsure	Yes	No	Unsure	Unsure	Unsure
Summary study attrition	Loss to follow-up is not associated with key characteristics, sufficient to limit potential bias of the observed relationship between PF and outcome	Moderate	High	Moderate	High	High	High	High

Domain	Issues for Consideration	Rating of Reporting						
		Gottlieb, et al. 1990	Eichhorn, et al. 1993	Madsen, et al. 1997	Cohen, et al. 2003	Corbi, et al. 2008	Adamopoulos, et al. 2009	Vaduganathan et al. 2013
3. Prognostic Factor (PF) Measurement								
3.1 Definition of the PF	A clear definition or description of serum Mg level or concentration	Yes	Yes	Unsure	Yes	Yes	Yes	Yes
3.2 Valid and reliable measurement of PF	Method of “serum Mg level or concentration” measurement is adequately valid and reliable to limit misclassification bias	Unsure	Yes	Unsure	Unsure	Unsure	Unsure	Yes
	Continuous variables are reported or appropriate cut points are used	Yes	Yes	Unsure	Yes	Yes	Unsure	No
3.3 Method and setting of PF measurement	The method and setting of measurement of “serum Mg level or concentration” is the same for all study participants	Unsure	Yes	Unsure	Yes	Yes	Yes	Yes
3.4 Proportion of data on PF available for analysis	Adequate proportion of the study sample has complete data for the “serum Mg level or concentration”	Yes	Yes	Unsure	Yes	Yes	Yes	Yes

3.5 Method used for missing data	Appropriate methods of imputation are used for missing “serum Mg level or concentration”	Unsure	Unsure	Unsure	Unsure	Unsure	Yes	Yes
PF measurement summary	PF is adequately measured in study participants to sufficient limit potential bias	High	Moderate	High	Moderate	Moderate	Moderate	Moderate

Domain	Issues for Consideration	Rating of Reporting						
		Gottlieb, et al. 1990	Eichhorn, et al. 1993	Madsen, et al. 1997	Cohen, et al. 2003	Corbi, et al. 2008	Adamopoulos, et al. 2009	Vaduganathan et al. 2013
4. Outcome Measurement								
4.1 Definition of the outcome	A clear definition or description of outcome is provided, including mortality (all-cause and cardiovascular-cause) and hospitalization	Unsure	Unsure	Yes	Unsure	No	No	Unsure
4.2 Valid and reliable measurement of outcome	Method of outcome measurement is adequately valid and reliable to limit misclassification bias	Yes	Yes	Yes	Yes	Yes	Unsure	Unsure
	Continuous outcome variables are reported, clearly or appropriate cut points are used	No	Yes	Yes	Yes	Unsure	Unsure	Unsure

4.3 Method and setting of outcome measurement	The method and setting of outcome measurement is the same for all study participants	Yes	Yes	Yes	Yes	Yes	Unsure	Yes
Outcome measurement summary	Outcome of interest is adequately measured in study participants to sufficiently limit potential bias	High	Moderate	Low	Moderate	High	High	High

Domain	Issues for Consideration	Rating of Reporting						
		Gottlieb, et al. 1990	Eichhorn, et al. 1993	Madsen, et al. 1997	Cohen, et al. 2003	Corbi, et al. 2008	Adamopoulos, et al. 2009	Vaduganathan et al. 2013
5. Study Confounding								
5.1 Important confounders measured	All important potential confounders are measured, including age, gender, Hypertension, Diabetes Mellitus, history of prior myocardial infarction, chronic kidney disease, NYHA functional class, LV ejection fraction (%)	Yes	Yes	Yes	Yes	Yes	Yes	Yes
5.2 Definition of the confounding factors	Clear definitions of the important confounders measured are provided	Unsure	Unsure	No	No	No	Unsure	Yes

5.3 Valid and reliable measurement of confounders	Measurement of all important confounders is adequately valid and reliable	No	Unsure	Unsure	No	Unsure	Unsure	Unsure
5.4 Method and setting of confounding measurement	The method and setting of confounding measurement are the same for all study participants	Unsure	Yes	Unsure	Unsure	Unsure	Unsure	Unsure
5.5 Method used for missing data	Appropriate methods are used if imputation is used for missing confounder data	Unsure	Unsure	Unsure	Unsure	No	Unsure	Yes
5.6 Appropriate accounting for confounding	Important potential confounders are accounted for in the study design and analysis (e.g. appropriate adjustment)	Unsure	Yes	Yes	Unsure	Unsure	Yes	Yes
Study confounding summary	Important potential confounders are appropriately accounted for, limiting potential bias with respect to the relationship between PF and outcome	High	High	High	High	High	High	Moderate

Domain	Issues for Consideration	Rating of Reporting						
		Gottlieb, et al. 1990	Eichhorn, et al. 1993	Madsen, et al. 1997	Cohen, et al. 2003	Corbi, et al. 2008	Adamopoulos, et al. 2009	Vaduganathan et al. 2013
6. Statistical Analysis and Reporting								
6. 1 Statistical Analysis and Reporting	There is sufficient presentation of data to assess the adequacy of the analysis	Unsure	Yes	Yes	Yes	Yes	Yes	Yes
6.2 Model development strategy	The strategy for model building (i.e., inclusion of variables in the statistical model) is appropriate and is based on a conceptual framework or model	Unsure	Unsure	Yes	Unsure	Unsure	Yes	Yes
	The selected statistical model is adequate for the design of the study	Yes	Unsure	Yes	Unsure	Yes	Yes	Yes
6.3 Reporting of results	There is no selective reporting of results	Unsure	Unsure	Yes	Yes	Yes	Yes	Yes
Statistical Analysis and presentation summary	The statistical analysis is appropriate for the design of the study, limiting potential for presentation of invalid or spurious results	High	High	Low	High	Moderate	Low	Low

Supplemental Digital Content 3. Patients' characteristics grouped according to serum magnesium concentration

Author	Year	N	Age (yr)			Serum Creatinine (µmol/l)			NYHA ≥ III (%)			Diuretic use (%)		
			Hypo mg	Normo mg	Hyper mg	Hypo mg	Normo mg	Hyper mg	Hypo mg	Normo mg	Hyper mg	Hypo mg	Normo mg	Hyper mg
Gottlieb [16]	1990	199	62.0±2	63.0±1	71.0 ±2†	185.6 ±26.5†	141.4 ±8.8	221± 17.7†	44.7	35.8	74.1†	…	…	…
Eichhorn [17]	1993	1068	60.0 ±12*	63.0 ±11	67.0 ±9†	114.9± 35.4†	123.8± 35.4	150.3± 44.2†	17.6*	6.2	22.3†	…	…	…
Cohen [11]	2003	404	70.8± 12.3*	72.6± 10.3	79.6± 5.3†	…	…	…	34.0	38.6	60.0†	…	…	…
Corbi [19]	2008	209	…	77.2± 7.01	77.0± 6.7†	…	97.2 ± 53.0	123.8†	…	…	…	…	60.3	50.0
Adamopoulos [20]	2009	1120	…	62.4± 10.7	65.1± 10.6†	…	106.1± 26.5	123.8± 35.4†	…	2.0	1.0	…	73.0	81.0†
Vaduganathan [12]	2013	1982	63.4± 11.7*	65.6± 11.9	67.7± 12.5†	106.1± 35.4†	…	150.3± 53.0†	45.0*	21.1	45.1†	97.8	96.4	97.6

Values reported as mean ± SD or count (%); …, data not available; mg, magnesemia

* Statistical significance ($P < 0.05$) of hypomagnesemic patients as compared with values in normomagnesemic patients

† Statistical significance ($P < 0.05$) of hypermagnesemic patients as compared with values in normomagnesemic patients

Supplemental Digital Content 4

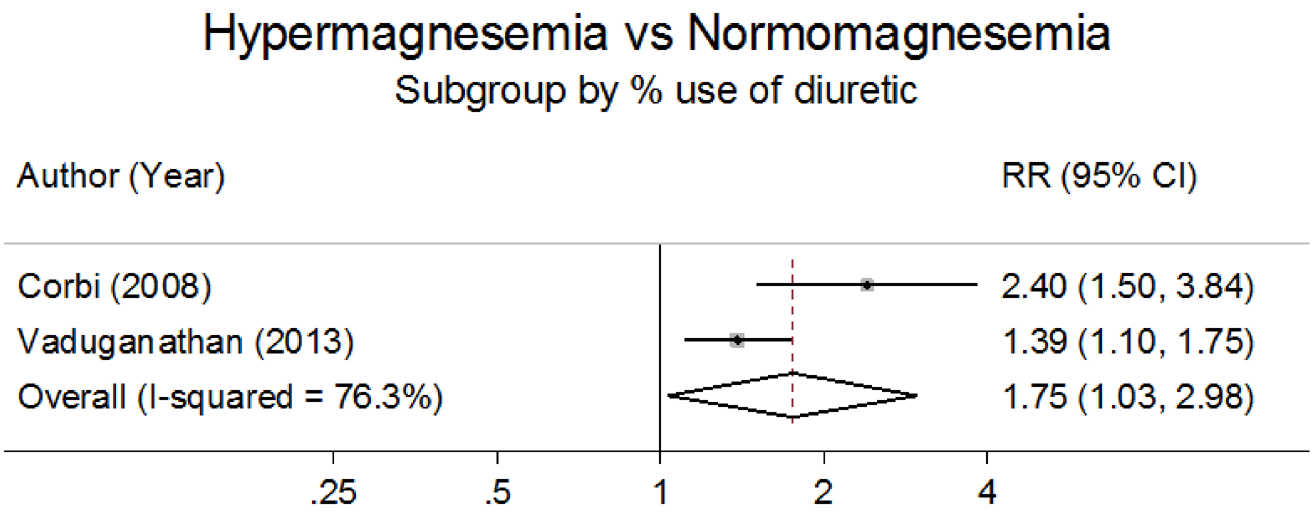

Supplemental Digital Content 5. Effects of hypermagnesemia vs normomagnesemia on cardiovascular mortality: sub-group analyses by factors

Subgroup	N	RR	95% CI	Q	I^2	*P*-value	Heterogeneity between subgroups *P*-value
Mean age							
≤ 70 y	2	2.04	0.81-5.19	4.43	77.4	0.035	0.250
> 70 y	4	1.29	1.06-1.56	2.14	0	0.544	
Follow up							
≤ 2 y	3	1.60	1.09-2.34	4.99	59.9	0.082	0.139
>2 y	3	1.07	0.75-1.53	0.71	0	0.700	
NYHA							
II-IV	2	1.24	0.92-1.68	1.72	42.0	0.189	0.158
Only III-IV	3	1.91	0.89-4.11	4.18	52.2	0.095	

NYHA indicates New York Heart Association (NYHA) functional classification

Supplemental Digital Content 6

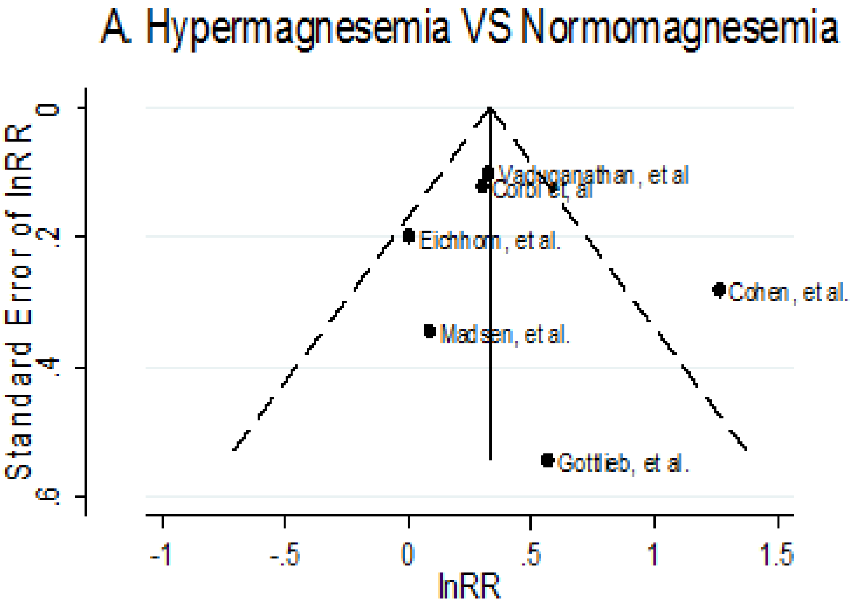

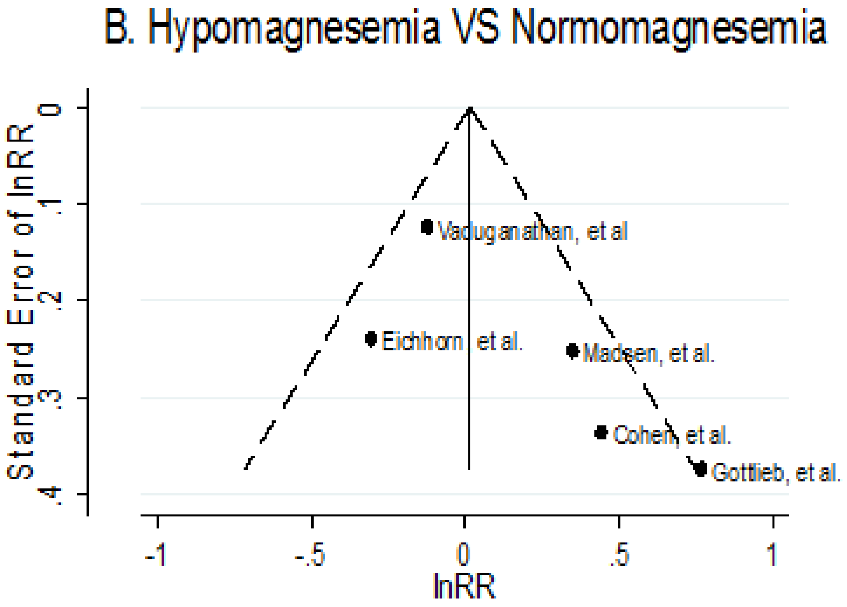

Supplemental Digital Content 7

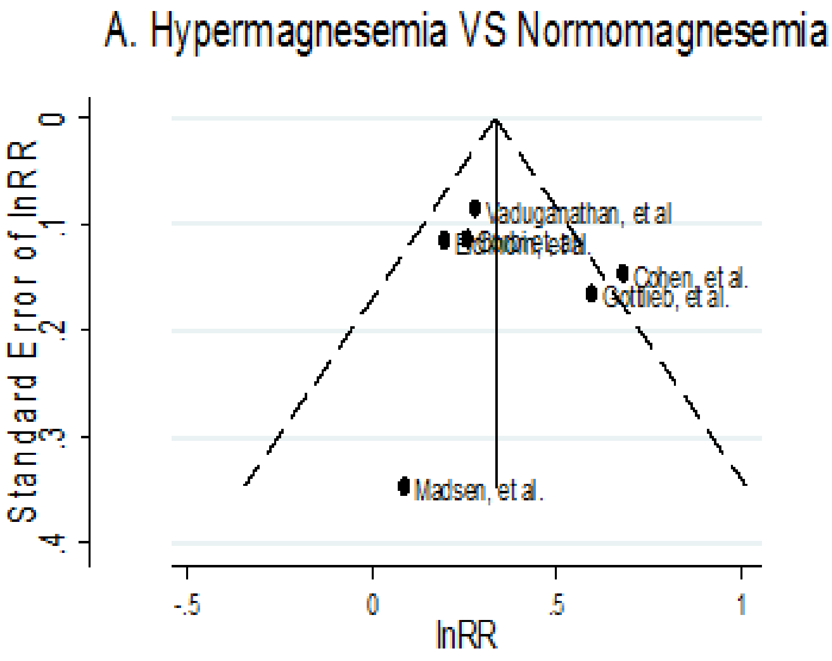

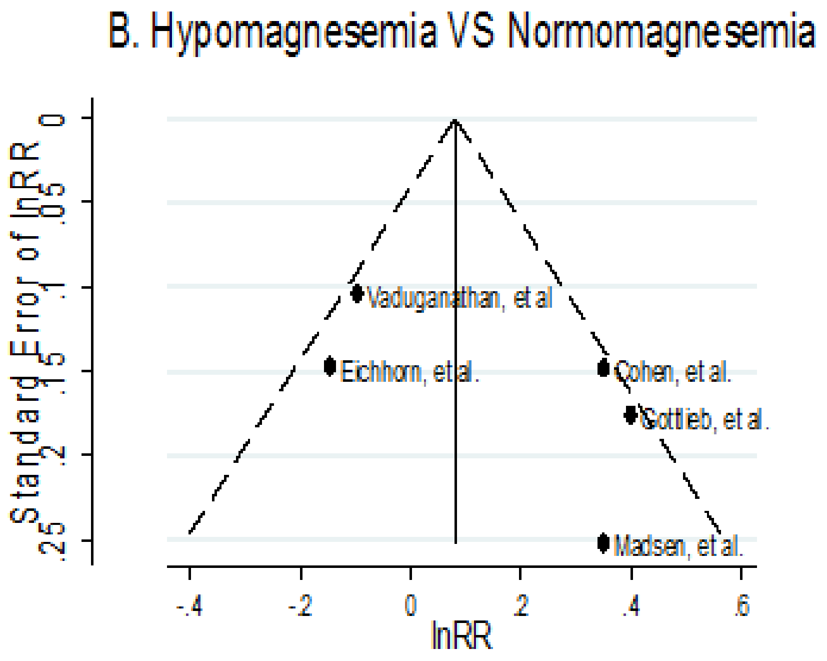

Supplement: Supplemental Digital Content [file md-95-e5406-s001.pdf]
